# Spontaneous retinal pigment epithelial tear in type 2 choroidal neovascularization: repair mechanisms following anti-VEGF therapy

**DOI:** 10.1186/s40942-019-0155-1

**Published:** 2019-01-23

**Authors:** Giuseppe Casalino, Vasuki Sivagnanavel, Samir Dowlut, Pearse A. Keane, Usha Chakravarthy

**Affiliations:** 1 Royal Eye Unit, Kingston Hospital NHS Foundation Trust, London, UK; 2 Moorfields Eye Hospital NHS Foundation Trust, London, UK; 3 Ophthalmology Macular Service, Belfast Health and Social Care Trust, Queen’s University of Belfast, Belfast, UK

**Keywords:** Age-related macular degeneration, Anti-VEGF therapy, Choroidal neovascularization, Multimodal retinal imaging, Repair mechanism, Retinal pigment epithelial tear

## Abstract

**Background:**

To report the clinical course and the multimodal retinal imaging of a spontaneous retinal pigment epithelial (RPE) tear in a type 2 choroidal neovascularization (CNV) secondary to age-related macular degeneration treated with anti-vascular endothelial growth factor (VEGF) treatment.

**Case presentation:**

A 74 year-old man presented with visual acuity deterioration in the right eye. Multimodal retinal imaging showed a predominantly classic (type 2) CNV complicated by a spontaneous RPE tear. The patient received six intravitreal injections of anti-VEGF which resulted in improvement of the vision and stabilization of the neovascular lesion on optical coherence tomography (OCT). Longitudinal changes of the RPE-photoreceptors interface, including RPE splitting, are reported on OCT.

**Conclusion:**

RPE tears may spontaneously occur in type 2 CNV. Anti-VEGF treatment should be aimed at promoting RPE repair and limiting the extent of the scarring. The mechanisms of RPE repair during treatment may be documented with OCT.

## Background

Retinal pigment epithelial (RPE) tears are a well recognised complication of neovascular age-related macular degeneration (nAMD) [1]; they can occur spontaneously or follow treatment with either anti-vascular endothelial growth factor (VEGF) agents or laser treatment, such as photodynamic therapy [1].

RPE tears have been mainly associated with the presence of a pigment epithelial detachment (PED) in the setting of different neovascular subtypes of AMD [1].

To the best of our knowledge, RPE tears have not been reported to occur during the natural history of type 2 (predominantly classic) choroidal neovascularizations (CNVs) secondary to AMD.

We report the clinical course and the multimodal retinal imaging of a spontaneous RPE tear in a type 2 CNV which was subsequently treated with anti-VEGF treatment.

## Case presentation

**Fig. 1:**
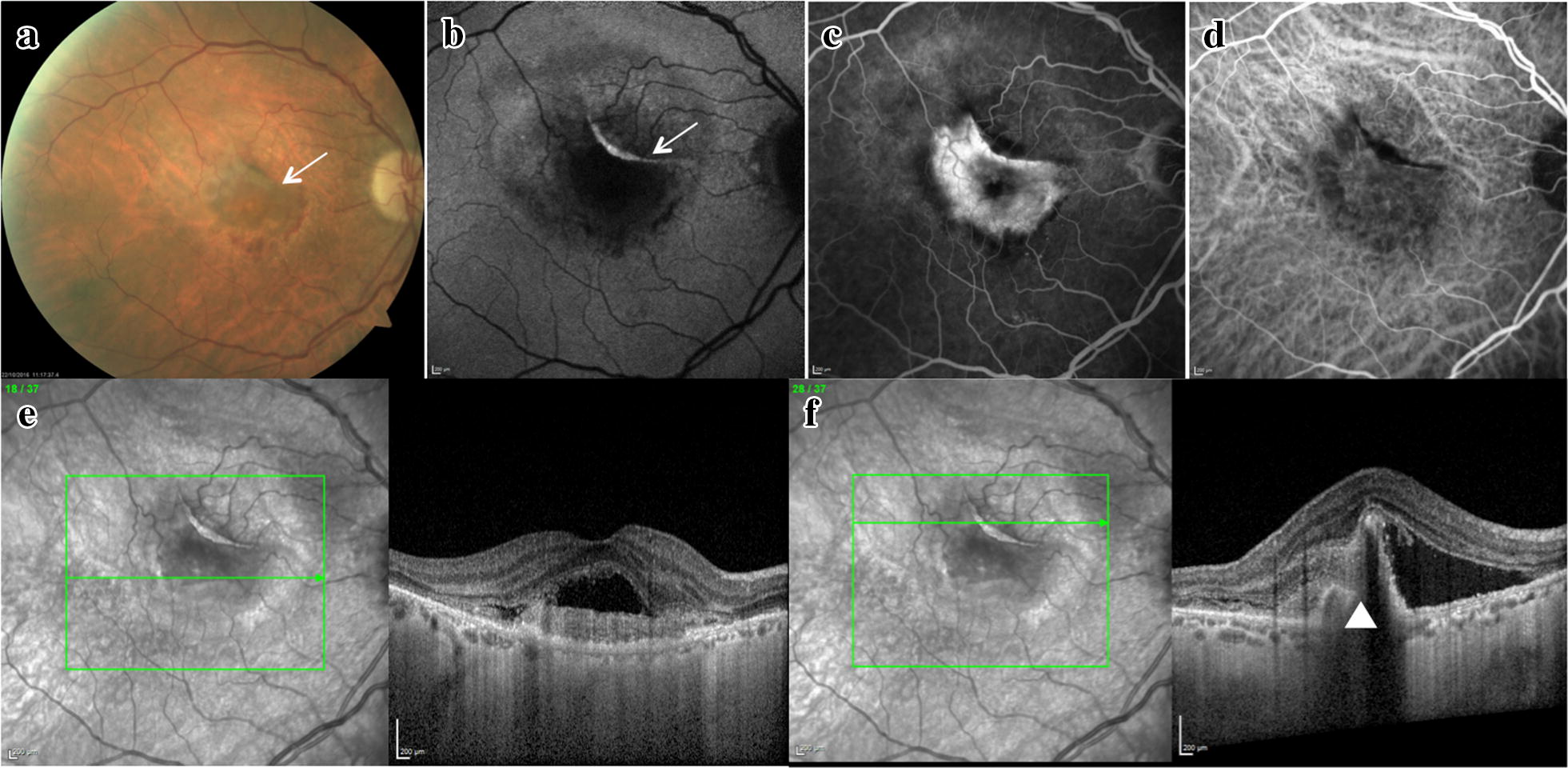
Multimodal retinal imaging of a spontaneous retinal pigment epithelium (RPE) tear in the right eye. **a** Color fundus photograph shows swelling and retinal haemorrhage at the macula and folding of the RPE superior to the fovea (arrow). **b** Fundus autofluorescence (AF) shows a hyper AF line in correspondence of the folded RPE (arrow). **c**, **d** Fluorescein angiography and indocyanine green angiography show presence of a predominanlty classic choroidal neovascularization (CNV). **e** Subfoveal optical coherence tomography (OCT) scan shows well defined subreitnal hyperreflective material and subretinal fluid. **f** OCT scan superior to the fovea shows interruption of the RPE (arrowhead)

**Fig. 2:**
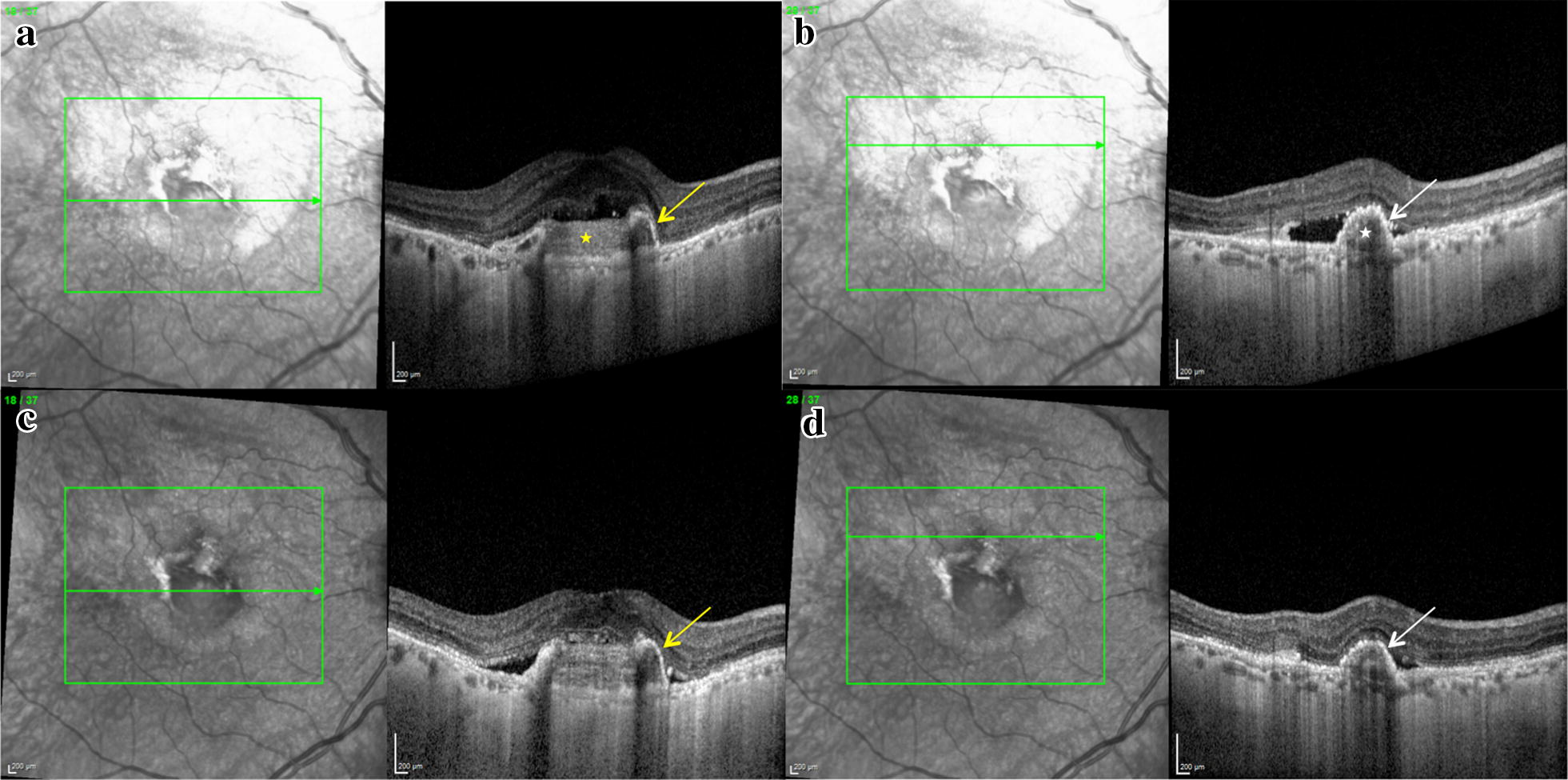
Optical coherence tomography (OCT) scan showing retinal pigment epithelium (RPE) repair process in the right eye during anti-VEGF treatment. 1 month after treatment: **a** subfoveal OCT scan shows splitting of the RPE (yellow arrow) with persistent well defined subretinal hyperreflective material (yellow star) and subretinal fluid; **b** OCT scan superior to the fovea shows continuity of the hyperreflective line attributable to the RPE (white arrow) and envelopment of the hyperreflective material (white star). 9 months after initiation of treatment; **c** Subfoveal OCT scan shows further consolidation of the hyperreflective material with persistent splitting of the RPE (yellow arrow); **d** OCT scan superior to the fovea shows reducing subretinal fluid and integrity of the RPE (white arrow)

A 74 year-old man presented with visual acuity deterioration in the right eye (RE). He had history of treated systemic hypertension. Best-corrected visual acuity (BCVA) was 20/80 in the RE and 20/20 in the LE. Fundus examination revealed a subretinal haemorrhage in the RE. Multimodal retinal imaging of the RE showed a type 2 (predominantly classic) CNV complicated by a spontaneous retinal pigment epithelial (RPE) tear (Fig. 1). Patient had not received any prior treatments. A course of 3 monthly intravitreal injections of Ranibizumab (0.5 mg × 0.05 mL) was administered. 4 weeks after the third injection OCT scan showed splitting and restoration of the hyperreflective line attributable to the RPE (Fig. 2). 9 months after initiation of treatment patient had received six intravitreal injections of Ranibizumab and BCVA improved to 20/32 in the RE.

## Discussion

RPE tears have been reported as part of the natural history of nAMD [2, 3]. Nowadays RPE tears are more commonly encountered after anti-VEGF treatment, although their incidence is relatively rare [4, 5].

Pathogenesis and repair mechanisms of RPE tears have not been fully elucidated. Established risk factors for RPE tear development are presence of PED, height of the PED and contracting folds in the RPE contour [6]. Anti-VEGF treatment seems to increase the risk of early tearing, possibly by shrinking the neovascular complex, thereby stretching the RPE to the breaking point [6].

Of note our patient presented with a type 2 (predominantly classic) CNV. RPE tears are extremely rare in type 2 CNVs and only two studies documented RPE tears occurring after anti-VEGF treatment in this subtype of CNV [7, 8]. However to the best of our knowledge RPE tears have not been documented during the natural history of type 2 CNVs secondary to AMD. Thus we report for the first time the multimodal retinal imaging of a spontaneous RPE tear in a type 2 CNV and we report repair mechanisms following anti-VEGF treatment.

In 1984 Gass [9] used fundus examinations and fluorescein angiography to describe the natural history and the tissue remodelling occurring in RPE tears secondary to nAMD. He hypothesized regrowth of hypopigmented pigment epithelium in the area where RPE was absent. 2 years later Chuang and Bird [10] supported Gass observations by reporting that in most cases the RPE defect was replaced by a plaque of fibrous tissue; in a few lesions the inner surface of Bruch’s membrane was observed to become covered by tissue with morphological features similar to the normal RPE [10].

The advent of high resolution OCT has made it possible to study the RPE-photoreceptors interface in more details. Recently Mukai et al. [11]. reported the longitudinal changes of the RPE tears on high resolution OCT and their findings supported the previously observed RPE remodelling [9, 10]. They observed that persistent subretinal fluid was associated with the repair process leading to fibrous tissue, while early subretinal fluid resolution was associated with the repair process leading to proliferative tissue ingrowth along Bruch’s membrane [11].

These observations have therapeutical implications and outline that when a RPE tear occur, regular treatment may stop the CNV from leaking and limit the retinal scarring [11]. Of note, in a recent study [12] it has been shown that the long-term visual outcome in eyes affected by RPE tears seems to be related more to the patient’s response to therapy than to the tear itself.

Recent multimodal retinal imaging studies showed ‘in vivo’ longitudinal plasticity of the RPE during anti-VEGF treatment of different subtypes of nAMD [13, 14]. An interesting reported finding is the splitting of the RPE into two distinct hyperreflective bands followed by the envelopment of the hyperreflective neovascular lesion on the OCT scan [14]. This latter finding is most commonly observed in type 2 CNV [14, 15] and indicates dynamic repair mechanisms of the RPE in the context of the subretinal neovascular lesion. Indeed it is known that in response to various stimuli, RPE cells are capable of hypertrophy, proliferation, and intraretinal migration. Such changes are commonly seen in AMD and have been reported on both high-resolution OCT and histologic sections [16, 17].

In line with the previous studies [13–15], our patient’s OCT scan showed prompt splitting of the hyperreflective line attributable to the RPE and envelopment of the hyperreflective neovascular lesion after anti-VEGF treatment.

In summary, RPE tears may spontaneously occur in type 2 CNV secondary to AMD. In such scenario, our case indicates that anti-VEGF treatment should be aimed at promoting RPE repair mechanisms and limiting the extent of the scarring. The mechanisms of RPE repair during treatment may be documented with OCT.
